# NIST System for Measuring the Directivity Index of Hearing Aids under Simulated Real-Ear Conditions

**DOI:** 10.6028/jres.118.005

**Published:** 2013-01-23

**Authors:** Randall P Wagner

**Affiliations:** National Institute of Standards and Technology, Gaithersburg, MD 20899-8223

**Keywords:** acoustical test manikins, directional hearing aids, directional response of hearing aids, hearing aid directivity index, hearing aid directivity measurements, hearing aid standards, manikin measurements, simulated real-ear conditions

## Abstract

The directivity index is a parameter that is commonly used to characterize the performance of directional hearing aids, and is determined from the measured directional response. Since this response is different for a hearing aid worn on a person as compared to when it is in a free field, directivity index measurements of hearing aids are usually done under simulated real-ear conditions. Details are provided regarding the NIST system for measuring the hearing aid directivity index under these conditions and how this system is used to implement a standardized procedure for performing such measurements. This procedure involves a sampling method that utilizes sound source locations distributed in a semi-aligned zone array on an imaginary spherical surface surrounding a standardized acoustical test manikin. The capabilities of the system were demonstrated over the frequency range of one-third-octave bands with center frequencies from 200 Hz to 8000 Hz through NIST participation in an interlaboratory comparison. This comparison was conducted between eight different laboratories of members of Working Group S3/WG48, Hearing Aids, established by Accredited Standards Committee S3, Bioacoustics, which is administered by the Acoustical Society of America and accredited by the American National Standards Institute. Directivity measurements were made for a total of six programmed memories in two different hearing aids and for the unaided manikin with the manikin right pinna accompanying the aids. Omnidirectional, cardioid, and bidirectional response patterns were measured. Results are presented comparing the NIST data with the reference values calculated from the data reported by all participating laboratories.

## 1. Introduction

Individuals with sensorineural hearing loss have significant difficulty understanding speech in the presence of background noise [1,2]. Hearing aids with directional microphones provide benefits, such as improvements in the signal to noise ratio, which can help remedy this problem [3–9]. Since the benefits provided are dependent on the directional performance characteristics of these hearing aids, measurement methods have been developed to assess these characteristics.

One parameter commonly used to quantify the directionality of an electroacoustic device that receives sound (sound receiver), such as a hearing aid, is the directivity index *D*. This parameter is frequency dependent, expressed in decibels (dB), and is given by

(1) $$D=10Log_{10}Q,$$

where *Q* is the directivity factor. This factor is expressed as

(2) $$Q=\frac{4\pi |M_{\mathrm{ax}}{|}^{2}}{\int_{0}^{2\pi }\int_{0}^{\pi }|M(\alpha ,\theta ){|}^{2}|\sin\theta |d\theta d\alpha },$$

where |*M_ax_*|^2^ is the magnitude of the mean-square sensitivity to sound waves that arrive at the device along the chosen reference axis, and |*M*(*α,θ*)|^2^ is the mean- square sensitivity to sound waves that arrive at the device from an elevation angle *α* and an azimuth angle *θ*. These equations are adapted from those originally presented to calculate *D* and *Q* for sound sources [10]. The only difference between the equations applicable to a sound source and those applicable to a sound receiver is that for a sound source, the sound pressure produced by the sound source is used in place of the sensitivity of the sound receiver.

The directivity index quantifies the directivity by effectively comparing the reference-axis sensitivity of the device to the sensitivity of the device to sound arriving from all directions. While the latter sensitivity can be measured in a diffuse sound field produced in a reverberant room, for practical reasons, this sensitivity is usually not determined in this manner. Instead, a diffuse sound field is simulated in an anechoic room by rotating the device under test and/or moving a sound source so that the source is sequentially used at different locations relative to the device with respect to the azimuth and elevation angles at equal distances from it. Alternatively, sound sources in an array can be used sequentially to simulate the diffuse field. Using either a single movable sound source or an array, data are acquired for each sound source location to measure the directional response and determine *Q*. When the directional response pattern is highly symmetric about the reference axis, which is commonly the case for directional microphones isolated in a free field, *Q* can be determined from data obtained only in the horizontal plane at *α* = 0° between the azimuth angles of 0° and 180° [11,12]. However, for directional response patterns that are asymmetric about the reference axis, such as those that result from the measurement conditions discussed herein, a different sampling method is required.

## 2. Measurement Method

Measurements of hearing aid characteristics under simulated real-ear conditions are done with standardized acoustical test manikins [13,14] to include the acoustical effects of a simulated median adult wearer on hearing aid performance. Procedures for such measurements are specified in U.S. national [15] and international [16] standards. For these measurement conditions, the directional response patterns of the hearing aids tested are considerably asymmetric about the reference axis. As is the case with an actual person, the head, torso and pinna of the manikin affect the directional response pattern and contribute to the asymmetry, as does the hearing aid style (in-the-ear, behind-the-ear, etc.) due to the location of the hearing aid microphone(s). Directivity index measurements of hearing aids are made at NIST according to the general method specified in the relevant U.S. national standard [15]. Directional response data are obtained using multiple sound source locations that are specified in terms of elevation and azimuth angles and distributed in a semi-aligned zone array on an imaginary spherical surface surrounding the manikin. Table 1 lists the azimuth and elevation angles for the forty eight sound source locations used in the minimal array specified in the standard.

**Fig. 1:**
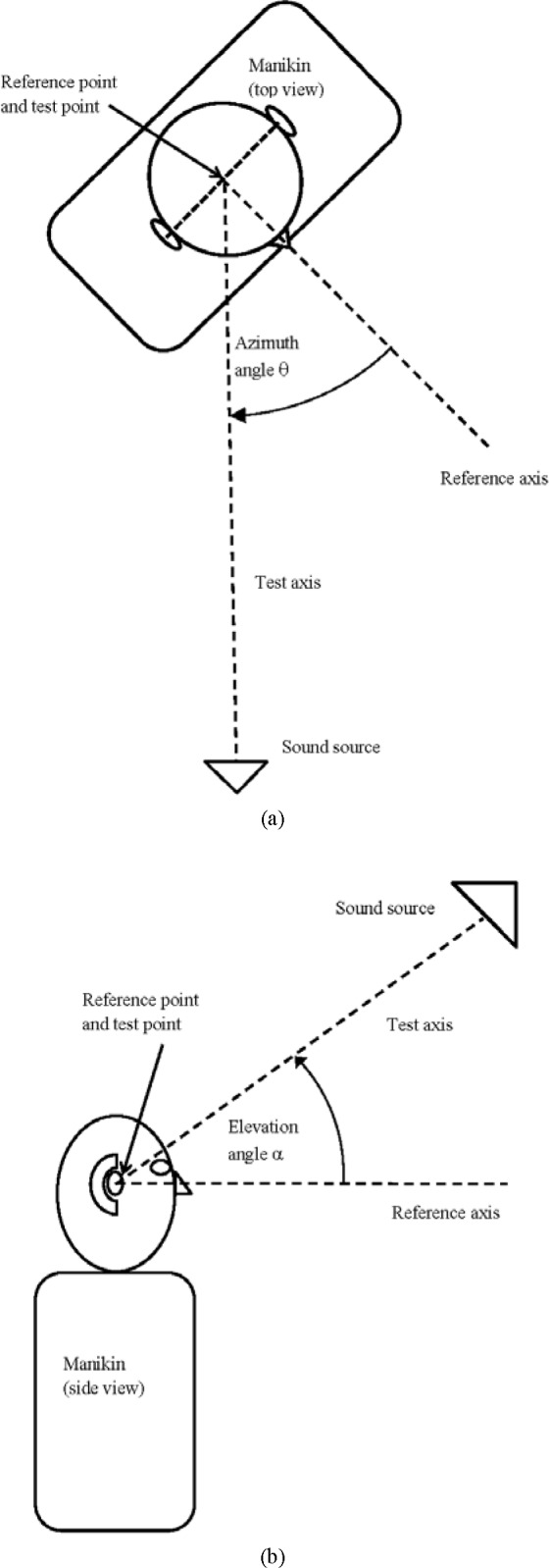
(a) Top view of the measurement system setup showing how the azimuth angle is defined, and (b) side view of the measurement system setup showing how the elevation angle is defined. (Not to scale)

Figure 1a shows the top view and Fig. 1b shows the side view of the measurement system setup and how the azimuth and elevation angles are defined between the reference axis of the manikin, which is mounted vertically in a position corresponding to that of a standing person, and the test axis of the sound source. The reference axis originates at the midpoint of the line joining the centers of the ear canal openings, known as the reference point of the manikin, and is normal to the vertical plane that contains this line. The test axis originates at the test point, which is the point in the test space where the reference point of the manikin is located during testing. This axis joins the test point and the center of the sound source. When the reference point of the manikin coincides with the test point, and the test axis coincides with the reference axis of the manikin (*α* = 0° and *θ* = 0°), the manikin is in the reference position and the sound source is located at what will be referred to here as the reference axis location. All sound source locations have a distance of 1.00 m ± 0.05 m from the test point.

Directional response data are acquired by making a series of sound pressure level (SPL) measurements as a function of frequency in one-third-octave band averages. All SPLs measured are expressed in dB with a reference sound pressure of 20 μPa. First, with the manikin absent, the free field SPL is determined at the test point as a function of source elevation angle. After the manikin is placed in the reference position, the hearing aid is placed on/in the ear of the manikin. Next, using a microphone threaded into the manikin ear simulator, the ear simulator SPL is measured sequentially for the reference axis location and for all other source locations. Finally, the ear simulator SPL measurement for the reference axis location is repeated. Directional response data for the open ear (manikin unaided) configuration are acquired in the same manner, but without any hearing aid in place.

For each given sound source location, the free field SPL measured for the applicable angle of elevation is subtracted from the ear simulator SPL measured for that sound source location to calculate a gain. The gain determined in this manner with a hearing aid in/on the manikin ear is referred to as the simulated real-ear aided gain, and for the open ear configuration the gain is called the manikin unaided gain. For the reference axis location, the gain is calculated using the average of the two simulator SPL measurements done with the sound source at this location [15]. The value of the directional response *R* (*α, θ*), expressed in decibels, for a given sound source location is equal to the gain determined for that location minus the gain determined for the reference axis location. When the directivity index measurement is implemented with the semi-aligned zone array sampling method that utilizes the forty eight sound source locations specified in the relevant U.S. national standard [15] and described here, Eq. (1) is rewritten as

(3) $$D=10Log_{10}{\left[\sum_{i=1}^{5}\frac{A_{i}}{n_{i}}\sum_{j=1}^{n_{i}}10^{R(\alpha_{i},\theta_{\mathrm{ij}})/10}\right]}^{-1},$$

where *i* is an integer for indexing the elevation angles, *A_i_* is the power weight of the elevation angle indexed by *i*, *n_i_* is the number of sound source locations at the elevation angle indexed by *i*, and *j* is an integer for indexing the azimuth angles. Table 2 lists the values of *i* and *A_i_* for each angle of elevation. The latter are standardized values [15] derived from calculations that account for the relative areas on the imaginary spherical surface associated with the sound source locations at a given angle of elevation. The directivity index determined in this manner for a hearing aid under simulated real-ear conditions is referred to as the simulated real-ear aided directivity index, and the directivity index determined in this manner for the open ear configuration of the manikin is referred to as the manikin unaided directivity index.

## 3. Measurement System Setup

**Fig. 2:**
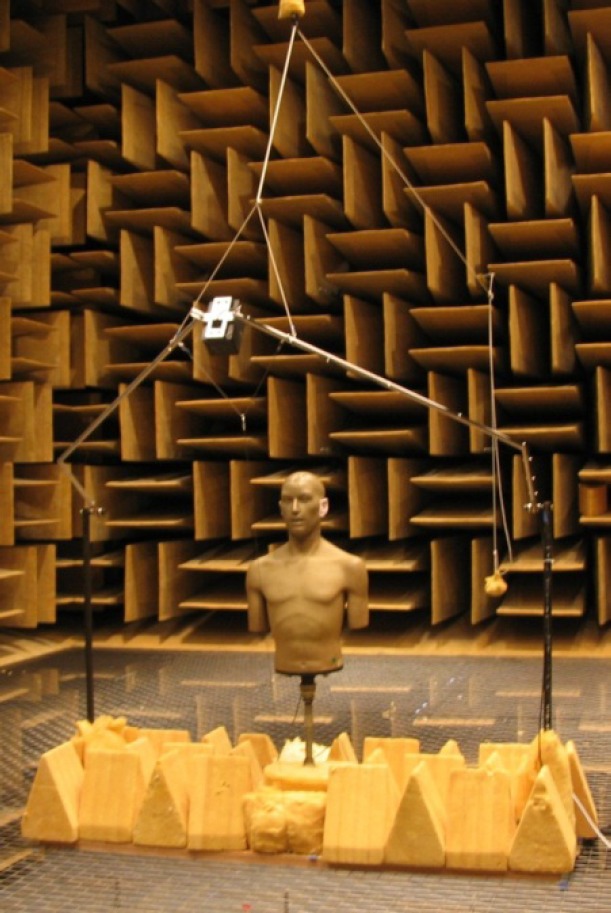
Photograph of the measurement system setup in the NIST large anechoic chamber. Hearing aids are tested in place on the manikin, which is mounted on a turntable that rotates the manikin in the horizontal plane to adjust the azimuth angle. Adjustment of the elevation angle is done with a vertical positioning system that moves a single loudspeaker on the path of a circular arc. A weight and pulley system elevates the loudspeaker, which is fixed at a given elevation angle by a thin steel stay wire.

Measurements are done in the NIST large anechoic chamber, which has acoustical properties that have been investigated and reported elsewhere [17]. The chamber free field dimensions, measured wedge tip to wedge tip, are 6.7 m by 10.0 m by 6.7 m. All interior surfaces of the chamber are covered with sound-absorptive wedge modules 1.8 m long that have a cutoff frequency of 45 Hz [18]. Figure 2 is a photograph of the measurement system setup in the anechoic chamber. A Knowles Electronics Manikin for Acoustics Research (KEMAR)^1^ [19], which meets the applicable specifications for standardized acoustical test manikins [13,14], is used with one neck ring in place and no clothing or wig. A standardized occluded-ear simulator [20] is used with the large pinnae supplied for KEMAR. Hearing aids are tested in/on the right ear of the manikin, which is mounted on a turntable that rotates the manikin in the horizontal plane to adjust the azimuth angle. The axis of rotation of the manikin passes through the reference point of the manikin. The manikin-turntable combination sits on a wooden support that is placed in the center of the steel wire mesh floor that spans the middle of the anechoic chamber. Both the turntable and the support are covered in fiberglass to absorb incident sound.

**Fig. 3:**
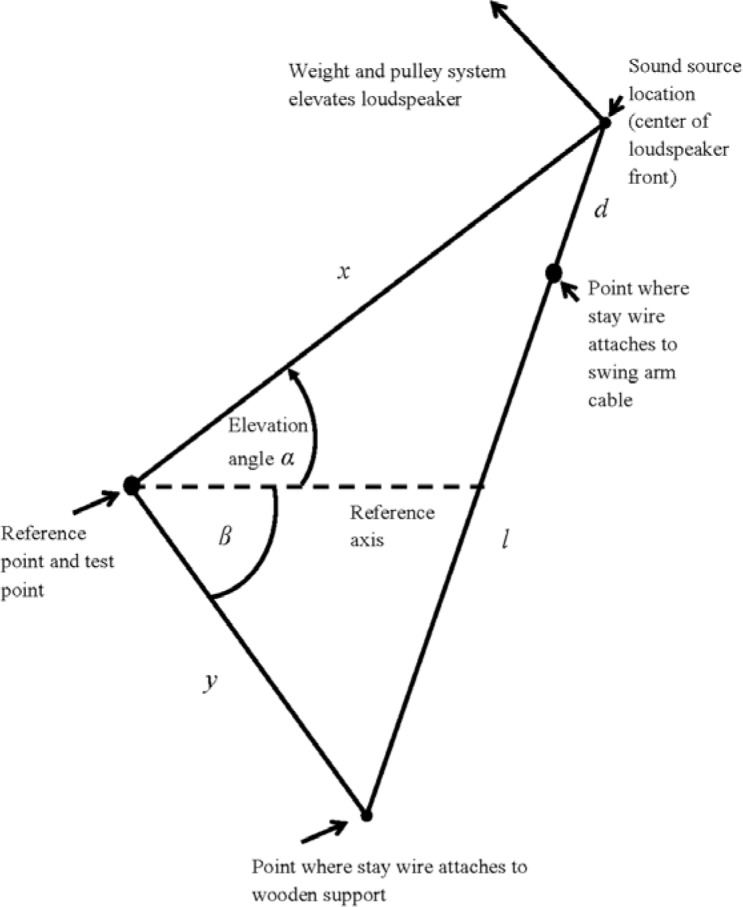
Geometric diagram that shows the triangle and the parameters used with the law of cosines to determine the stay wire length *l* required to set the sound source at a given elevation angle *α*. (Not to scale)

Adjustment of the elevation angle is done with a vertical positioning system that moves the loudspeaker used as the sound source on the path of a circular arc. The arc has a 1 m radius and is centered on the test point so that the 1 m distance required from the sound source to the test point is maintained at all elevation angles. The loudspeaker is mounted at the center of a swing arm, which is attached to poles fixed to separate ends of the wooden support. A weight and pulley system elevates the swing arm and loudspeaker, which is fixed at a given elevation angle by a thin steel stay wire attached to the wooden support at one end and to a cable fixed to the swing arm at the other end. The elevation angle is determined by the stay wire length *l*. By applying the law of cosines, the value of *l* required for each elevation angle was calculated using

(4) $$l={\left[x^{2}+y^{2}-2xy\cos(\alpha +\beta )\right]}^{\frac{1}{2}}-d,$$

where *x* is the distance from the test point to the sound source, *y* is the distance from the test point to where the stay wire attaches to the wooden support, *d* is the distance from the sound source to where the stay wire attaches to the swing arm cable, and *β* is a fixed angle. Figure 3 is a geometric diagram that shows the triangle used with the law of cosines and the parameters of Eq. (4).

Both the free field SPL and the simulator SPL are measured with a standardized International Electrotechnical Commission type WS2P microphone [21]. For the free field measurements, the principal axis of the microphone, which is the line through the center of and perpendicular to the microphone diaphragm, is perpendicular to the test axis so that the microphone is at grazing incidence to the incident sound regardless of elevation angle. A random noise signal with equal energy per unit Hertz and a crest factor of 12 dB is used as the test stimulus. The free field SPL used is normally 60 dB, although an SPL as high as 66 dB has been used to achieve a higher signal-to-noise ratio at low frequencies. The lower SPL is preferred since it usually provides an adequate signal-to-noise ratio and is less likely to result in compression of the hearing aid output signal. Data are obtained with a dynamic signal analyzer that uses Fast-Fourier-Transform (FFT) techniques. A Hann window function is applied during the FFT processing. The widest frequency range over which the system has been used to acquire data is the range of one-third-octave bands with center frequencies from 200 Hz to 8000 Hz. Data for one-third-octave bands with center frequencies from 200 Hz to 1250 Hz are acquired with 801 frequency lines from 0 Hz to 1562.5 Hz, and data for one-third-octave bands with center frequencies from 1600 Hz to 8000 Hz are acquired with 801 frequency lines from 0 Hz to 10 000 Hz.

## 4. Uncertainty of Measurement Results

Uncertainties of the measurement results obtained for each one-third-octave frequency band of measurement with the NIST system are shown for the directivity factor in Table 3, and for the directivity index in Table 4. These uncertainties were developed by applying established guidelines for evaluating and expressing uncertainties [22]. Standard uncertainties were combined to obtain expanded (coverage factor *k* = 2) uncertainties *U_N_* for the NIST results. All the standard uncertainties were determined from a Type B evaluation, done by assuming a symmetric rectangular (uniform) probability distribution. Values for the upper and lower bounds of the distribution were established by estimating the limits of the effects on the measurement results of a given source of uncertainty. The standard uncertainty due to each source of uncertainty was determined by calculating the standard deviation of the rectangular probability distribution, which is one half of the width of the distribution divided by the square root of three. Initial limits rounded to the nearest 0.1 dB were established for the directivity index. These limits were transformed to percentages and rounded to the nearest 0.1 % in order to calculate standard and expanded uncertainties expressed in percent (as relative uncertainties) for the directivity factors. These expanded uncertainties were transformed to logarithmic quantities to determine the expanded uncertainties for the directivity indices to the nearest 0.1 dB.

The standard uncertainties *u_1_* are due to limitations in the short term repeatability of the SPL measurements made with the dynamic signal analyzer. Limits used to calculate *u_1_* were determined from a series of repeated measurements done while acquiring free field SPL data at the test point with the manikin absent for the five elevation angles used in the measurements. The standard uncertainties *u_2_* arise from the differences between free field SPLs for the different elevation angles and were calculated from limits determined from the same series of repeated measurements. The standard uncertainties *u_3_* are due to the effects of noise on the measurements, mainly the acoustical noise produced by a hearing aid. Limits used to calculate *u_3_* were determined from measurements of the signal-to-noise ratio in the ear simulator done with hearing aids in place on the manikin and the sound source at the reference axis location. Relatively higher limits are assigned to the frequency bands below the band with a 400 Hz center frequency since low frequency hearing aid noise results in lower measured signal-to-noise ratios for those bands. The standard uncertainties *u_4_* are attributed to system and hearing aid gain drift that occurs during the period it takes to complete a full set of directional measurements. Limits used to calculate *u_4_* were determined from differences found between the data obtained at the beginning and at the end of this period with the sound source at the reference axis location. The standard uncertainties *u_5_* arise from limitations of the forty eight point semi-aligned zone array sampling method. Limits used to calculate *u_5_* were obtained from results reported for simulations and measurements done for second-order directional systems under simulated real-ear working conditions [15]. The standard uncertainties *u_6_* arise from imperfections in setting the elevation angle in combination with imprecise vertical aiming of the directional response pattern. Limits used to calculate *u_6_* were estimated from the results of calculations reported for a second-order directional system using an elevation angle misalignment of no more than ±2° for any of the elevation angles [15]. Results for second-order directional systems were used to estimate limits for determining *u_5_* and *u_6_* since this is the lowest order for which such results are given in the U.S. national standard for hearing aid measurements under simulated real-ear conditions [15]. Limits for the first-order systems discussed herein are expected to not exceed those of the second-order systems used to calculate *u_5_* and *u_6_*. When calculated without including *u_5_* and *u_6_*, none of the expanded uncertainties decrease by more than 0.1 dB. The standard uncertainties *u_7_* arise from effects on the measurements done with the sound source at the reference axis location due to misalignment of the directional response pattern. Frequency dependent limits used to calculate *u_7_* were determined using data acquired at the sound source locations of the forty eight point semi-aligned zone array immediately adjacent to the reference axis location in the vertical and horizontal planes. Component contributions due to a maximum potential misalignment of ±2° in the azimuth and elevation angles were calculated from the data and combined to determine these limits.

## 5. Overview of Interlaboratory Comparison

To determine the reproducibility of results obtained with the forty eight point semi-aligned zone array sampling method specified in the U.S. national standard for hearing aid measurements under simulated real-ear conditions [15], an interlaboratory comparison was performed in 2004 and 2005. Details regarding this comparison, along with some data and results obtained from it, have been reported elsewhere [23]. This comparison was conducted between eight different laboratories, including NIST, of members of Working Group S3/WG48, Hearing Aids, established by Accredited Standards Committee S3, Bioacoustics, which is administered by the Acoustical Society of America and accredited by the American National Standards Institute. The analysis reported herein is limited to a comparison of the NIST results with reference values computed by consolidating the results from all participating laboratories.

Two different programmable first-order directional hearing aids and a large right pinna for KEMAR were circulated among the laboratories. Both hearing aids had three different programmed memories, and were in-the-ear aids customized to fit the accompanying pinna. The first aid was designed and programmed so that the first memory had an omnidirectional response pattern, and the second and third memories had a cardioid response pattern. The second aid was designed and programmed so that the first memory had an omnidirectional response pattern, and the second and third memories had a bidirectional (dipole) response pattern. For both aids, the second memory had a low frequency gain boost as compared to the third memory. In addition to measuring the simulated real-ear aided directivity index for all six memories, each laboratory measured the manikin unaided directivity index using the pinna circulated between laboratories for the measurements. The protocol for the comparison called for data acquisition over the frequency range of one-third-octave bands with center frequencies from 500 Hz to 5000 Hz. An extended frequency range of one-third-octave bands with center frequencies from 200 Hz to 8000 Hz was optional.

**Fig. 4:**
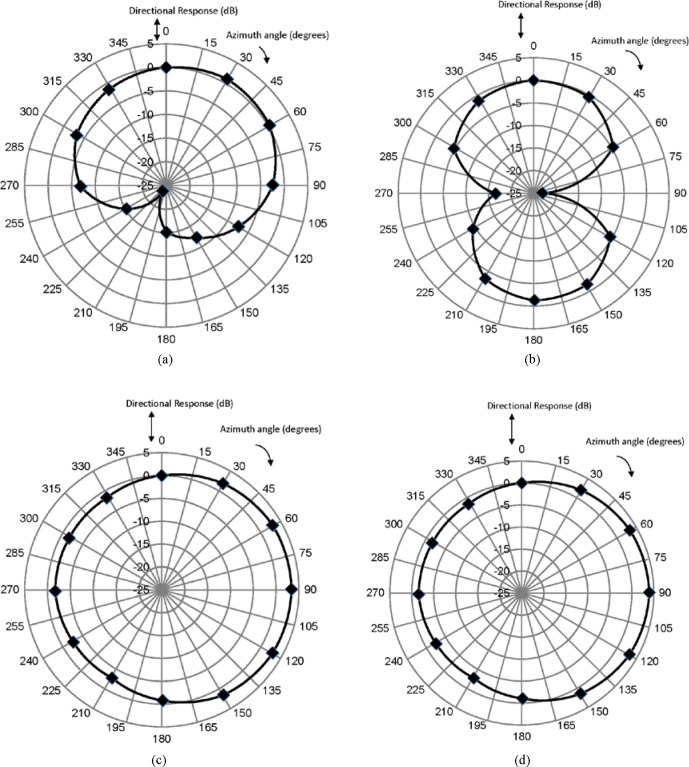
Polar response patterns measured at NIST in the horizontal plane at *α* = 0° for the one-third-octave band with a center frequency of 500 Hz. Measured data are shown only for azimuth angles included in the forty eight point semi-aligned zone array, the lines in between these angles were interpolated. Response data are displayed for (a) a hearing aid programmed with a cardioid pattern, (b) a hearing aid programmed with a bidirectional pattern, (c) a hearing aid programmed with an omnidirectional pattern (d) an unaided manikin.

To show examples of the directional response patterns programmed in the aids used in the inter-laboratory comparison and intrinsic to the unaided manikin, polar response patterns measured at NIST in the horizontal plane at *α* = 0° for three of the programmed memories and the unaided manikin are displayed in Fig. 4a through Fig. 4d. All these patterns were obtained for the one-third-octave band with a center frequency of 500 Hz. The figures display measured data only for azimuth angles included in the forty-eight point semi-aligned zone array. The lines in between these angles were interpolated. As expected, due to the simulated real-ear measurement conditions, all these response patterns appear asymmetric.

## 6. Analysis of NIST Results

The simulated real-ear directivity index for all six hearing aid memories and the manikin unaided directivity index were measured at NIST over the extended frequency range of the interlaboratory comparison. Results obtained for *Q* and *D* from these measurements will be denoted here as *Q_N_* and *D_N_*, respectively. In order to compare the NIST results with the combined results of all the laboratories that participated in the comparison, reference values *Q_ref_* and *D_ref_* were computed for the values of *Q* and *D*, respectively. For a given frequency band and memory (or the unaided manikin), *Q_ref_* was determined by calculating the unweighted mean of the comparison results for *Q*. Equation (1) was then used to calculate *D_ref_*. The expanded uncertainties *U_ref_* of the reference values *Q*_ref_ were calculated using

(5) $$U_{\mathrm{ref}}=\pm \frac{\left(t_{n-1}\times s\right)}{\sqrt{n}},$$

where *n* is the number of laboratories, *s* is the standard deviation of the comparison values, and *t_n-_*_1_ is the value of the Student *t*-distribution for *n*-1 degrees of freedom and a 95 % confidence level. These expanded uncertainties were transformed to logarithmic quantities to determine the expanded uncertainties for the values of *D_ref_*. Eight laboratories provided data for all of the one-third-octave bands except those with center frequencies of 200 Hz, 250 Hz, 315 Hz, 6300 Hz and 8000 Hz. For those bands, the number of participating laboratories was seven.

**Fig. 5:**
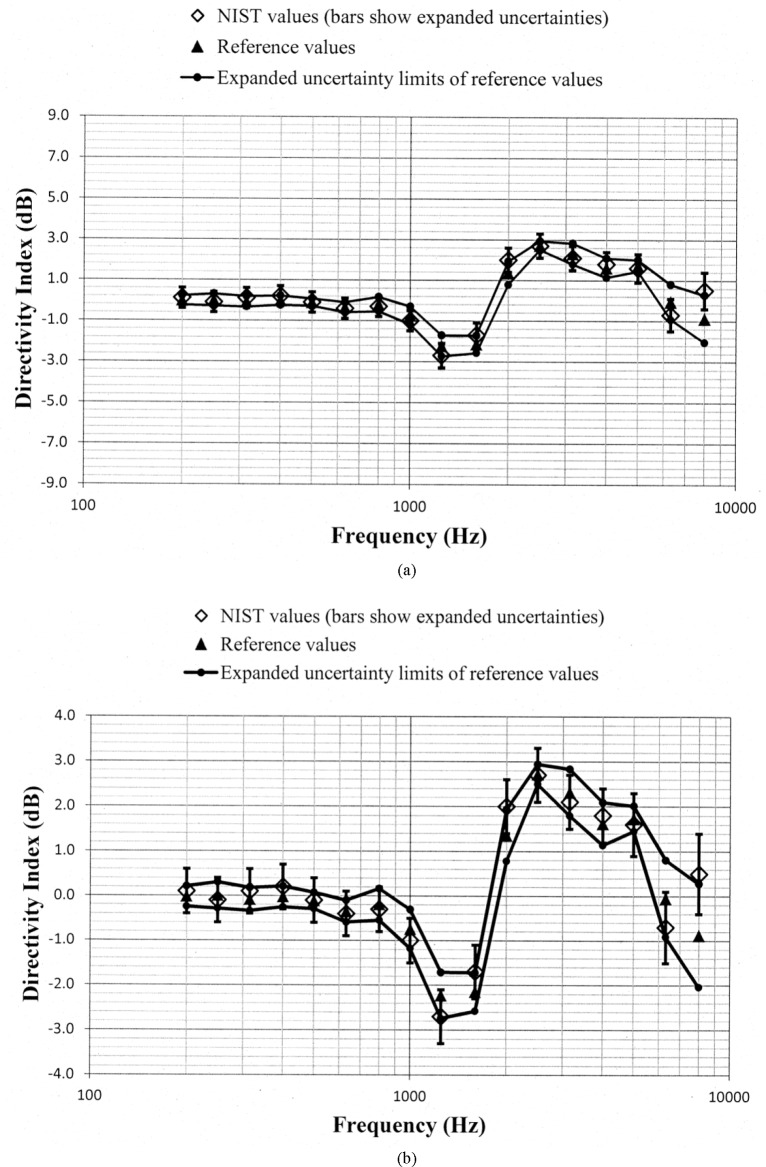
Comparison of the NIST values and the reference values for the simulated real-ear aided directivity index for the omnidirectional response pattern programmed in the first of the two aids circulated in the S3/WG48 interlaboratory comparison. These data are shown with (a) a vertical scale that is the same for Fig. 5a through Fig. 11a for easier comparison between figures, and with (b) an expanded vertical scale to more easily differentiate between the data displayed in the plot.

**Fig. 6:**
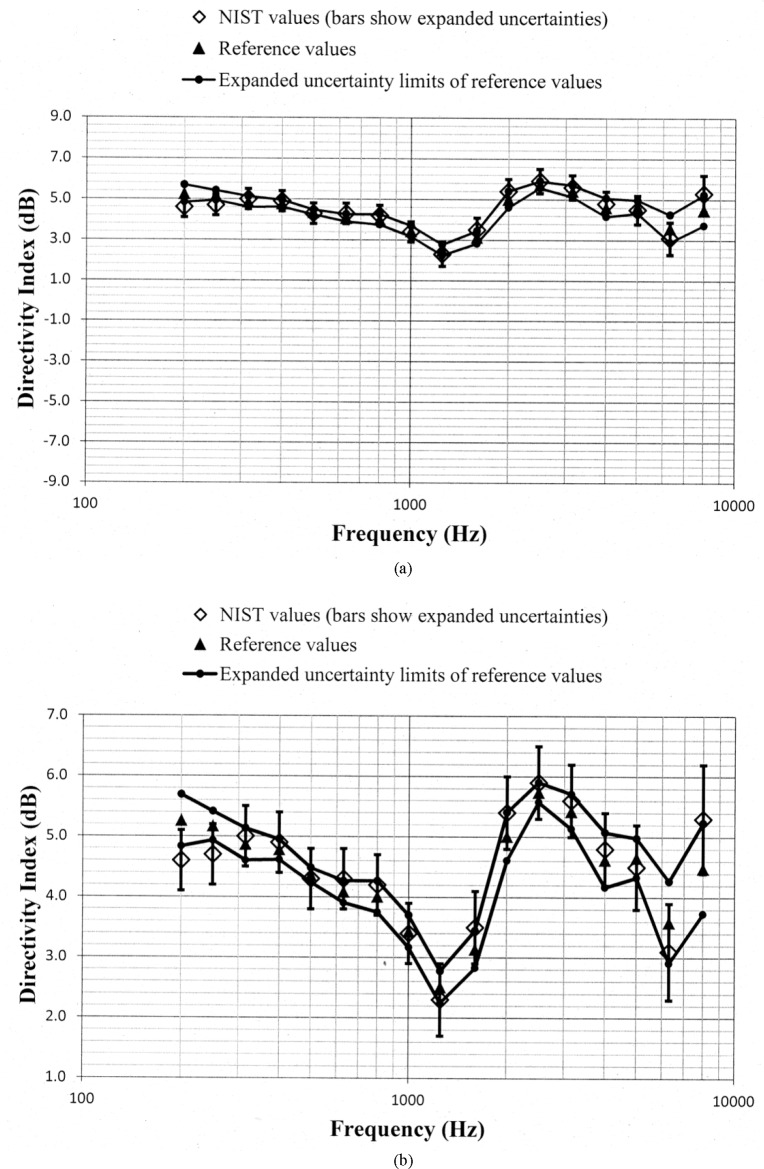
Comparison of the NIST values and the reference values for the simulated real-ear aided directivity index for the cardioid response pattern with low frequency gain boost programmed in the first of the two aids circulated in the S3/WG48 interlaboratory comparison. These data are shown with (a) a vertical scale that is the same for Fig. 5a through Fig. 11a for easier comparison between figures, and with (b) an expanded vertical scale to more easily differentiate between the data displayed in the plot.

**Fig. 7:**
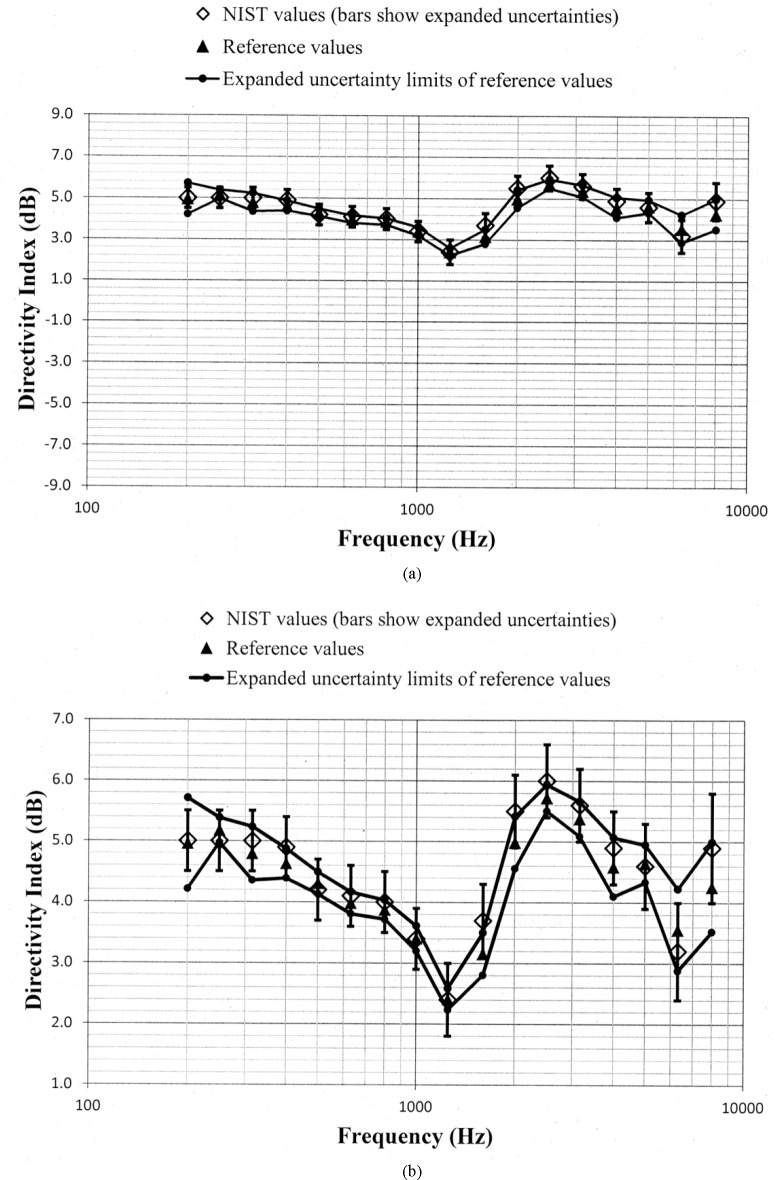
Comparison of the NIST values and the reference values for the simulated real-ear aided directivity index for the cardioid response pattern without low frequency gain boost programmed in the first of the two aids circulated in the S3/WG48 interlaboratory comparison. These data are shown with (a) a vertical scale that is the same for Fig. 5a through Fig. 11a for easier comparison between figures, and with (b) an expanded vertical scale to more easily differentiate between the data displayed in the plot.

**Fig. 8:**
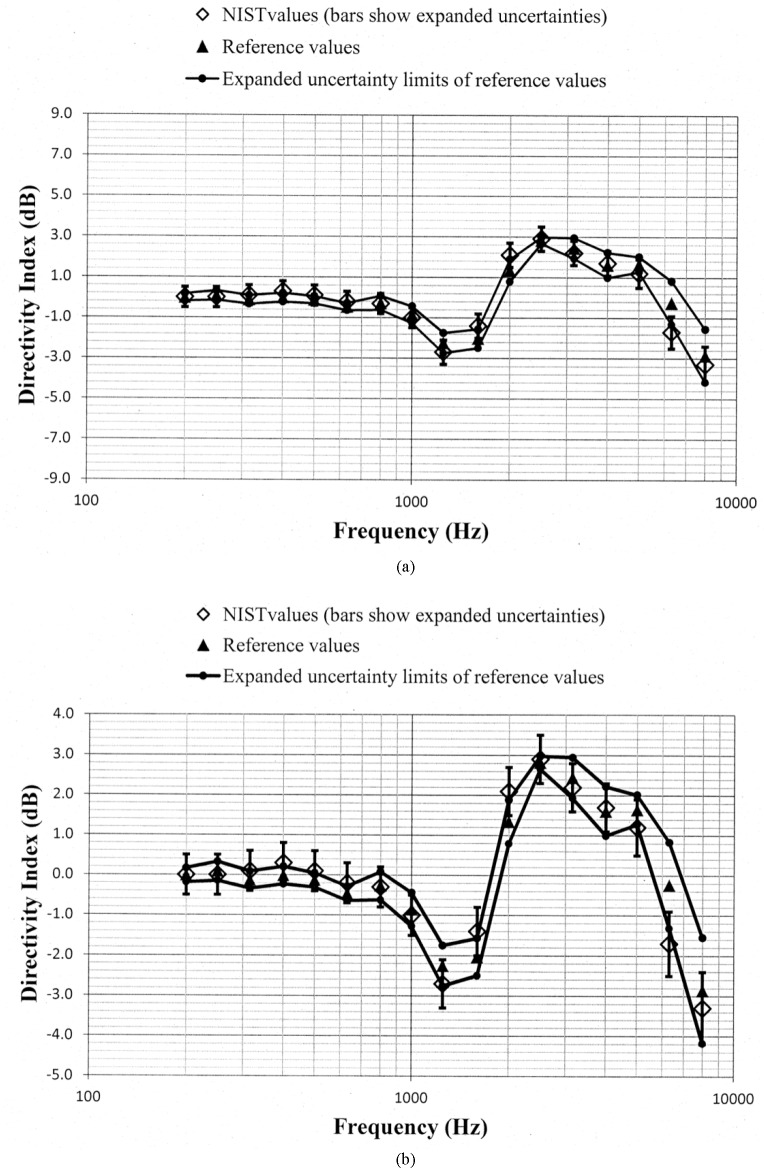
Comparison of the NIST values and the reference values for the simulated real-ear aided directivity index for the omnidirectional response pattern programmed in the second of the two aids circulated in the S3/WG48 interlaboratory comparison. These data are shown with (a) a vertical scale that is the same for Fig. 5a through Fig. 11a for easier comparison between figures, and with (b) an expanded vertical scale to more easily differentiate between the data displayed in the plot.

**Fig. 9:**
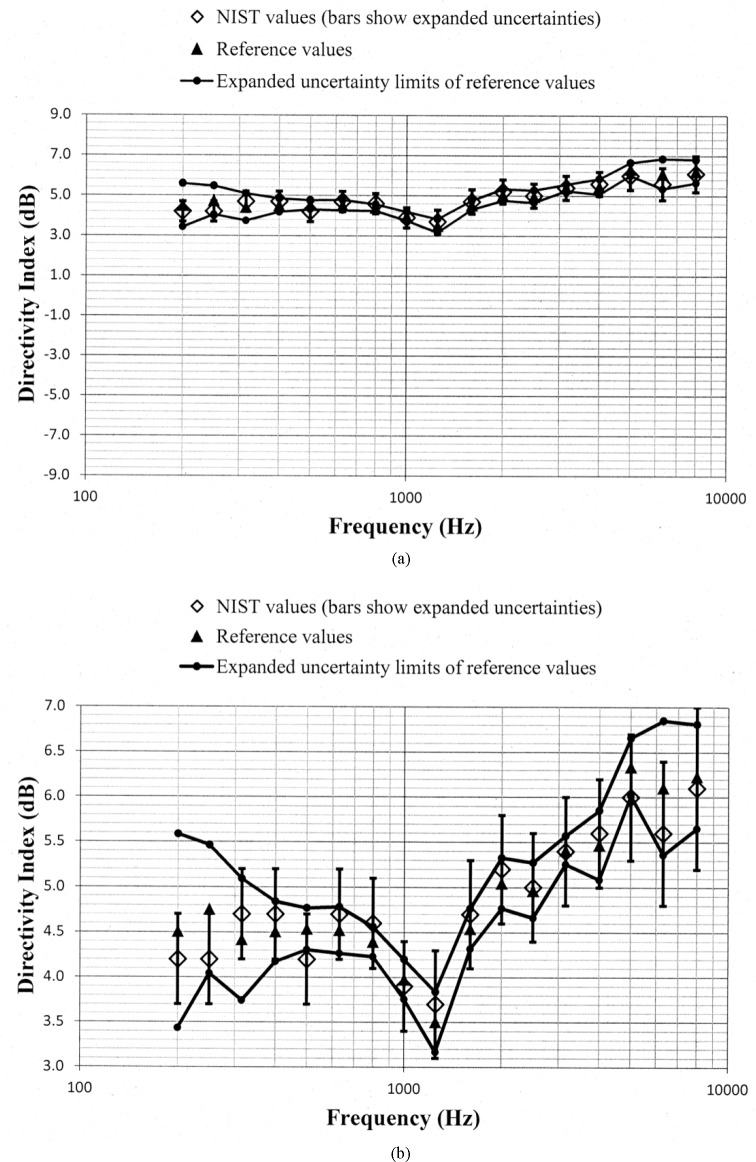
Comparison of the NIST values and the reference values for the simulated real-ear aided directivity index for the bidirectional response pattern with low frequency gain boost programmed in the second of the two aids circulated in the S3/WG48 interlaboratory comparison. These data are shown with (a) a vertical scale that is the same for Fig. 5a through Fig. 11a for easier comparison between figures, and with (b) an expanded vertical scale to more easily differentiate between the data displayed in the plot.

**Fig. 10:**
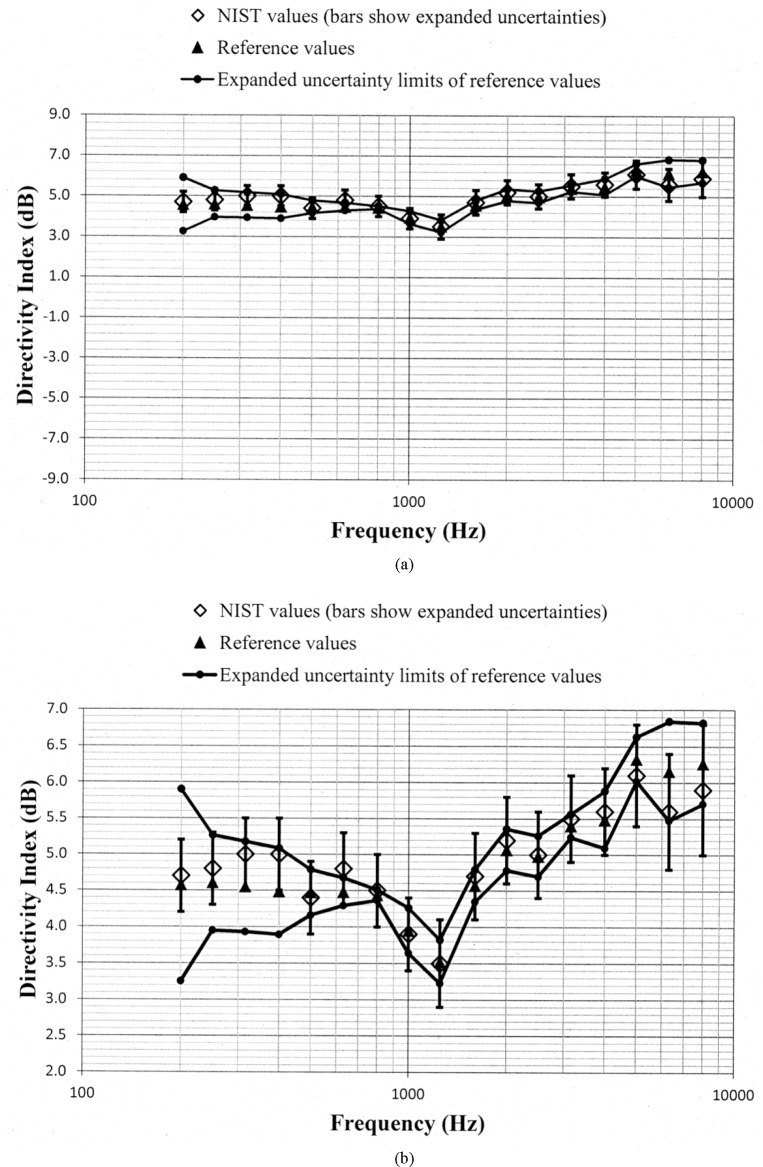
Comparison of the NIST values and the reference values for the simulated real-ear aided directivity index for the bidirectional response pattern without low frequency gain boost programmed in the second of the two aids circulated in the S3/WG48 interlaboratory comparison. These data are shown with (a) a vertical scale that is the same for Fig. 5a through Fig. 11a for easier comparison between figures, and with (b) an expanded vertical scale to more easily differentiate between the data displayed in the plot.

**Fig. 11:**
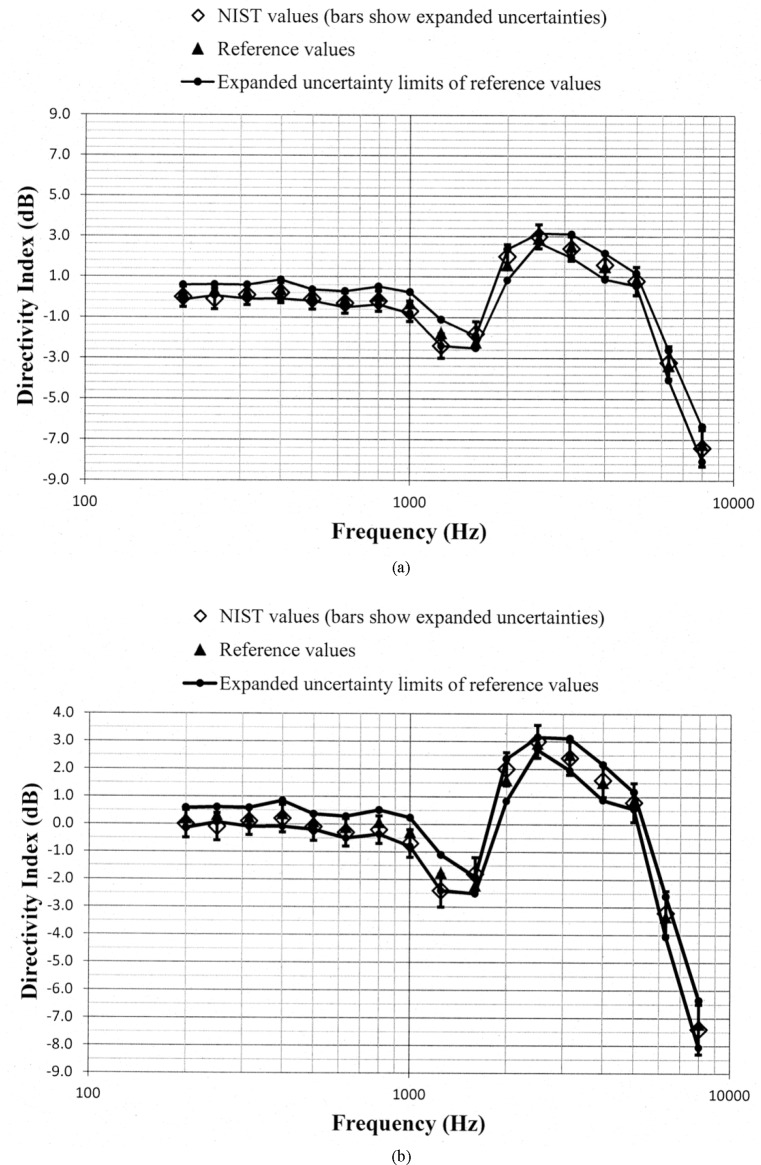
Comparison of the NIST values and the reference values for the manikin unaided directivity index for the manikin right pinna circulated in the S3/WG48 interlaboratory comparison. These data are shown with (a) a vertical scale that is the same for Fig. 5a through Fig. 11a for easier comparison between figures, and with (b) an expanded vertical scale to more easily differentiate between the data displayed in the plot.

**Fig. 12:**
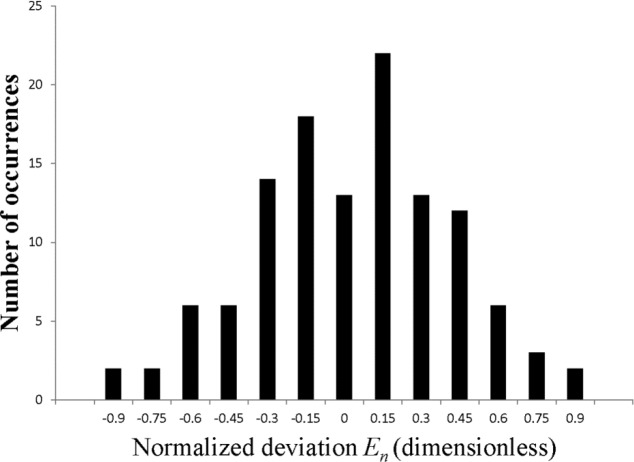
Histogram of all of the values of the normalized deviation *E_n_* calculated from the NIST results and the reference values for the directivity factor from the S3/WG48 interlaboratory comparison.

Values of *D_N_* and *D_ref_*, along with the uncertainty limits for these values, are shown as a function of frequency in Fig. 5 through Fig. 10 for each of the programmed memories and in Fig. 11 for the unaided manikin. The same vertical scale is used in Fig. 5a through Fig. 11a for easier comparison between figures. Expanded vertical scales are used in Fig. 5b through Fig. 11b to more easily differentiate between the data displayed in a given figure. Visual inspection of the plots indicates that values of *D_N_* and *D_ref_* track each other very closely with changes in frequency. Also, the uncertainty limits for these values consistently display some degree of overlap. Plots of the directivity indices in Fig. 5 and Fig. 8 for the omnidirectional response patterns programmed in the two different aids and the plot of the indices shown in Fig. 11 for the unaided manikin are very similar except at the higher frequencies. At any given frequency, lower values of the directivity indices are measured for these cases as compared to the indices shown in Fig. 6 and Fig. 7 for the cardioid response patterns and in Fig. 9 and Fig. 10 for the bidirectional response patterns. Comparisons of Fig. 6 with Fig. 7, and Fig. 9 with Fig. 10, indicate that low frequency gain boost does not have an effect on the directivity indices for either the cardioid or bidirectional response patterns. Results obtained with the NIST system were compared with the reference values using a conventional approach for judging the quality of measurement results obtained at a laboratory participating in an interlaboratory comparison [24]. A normalized deviation *E_n_* defined by

(6) $$E_{n}=\frac{Q_{N}-Q_{\mathrm{ref}}}{\sqrt{U_{N}^{2}+U_{\mathrm{ref}}^{2}}},$$

was calculated for each of the 119 values of *Q_N_* measured (seven directional response patterns as a function of seventeen frequency bands). A value of |*E_n_*| less than unity is required for the measurement result to be considered acceptable, effectively in agreement with the reference value. All of the NIST results obtained for the interlaboratory comparison met this criterion. A value of ∣*En*∣ greater than unity would indicate that the difference between the NIST result and the reference value is greater than what would be expected based on the combined uncertainties. Values of *E_n_* calculated for these results are displayed as a histogram in Fig. 12 and visually appear to be normally distributed. In order to test if the data can be considered to fit a normal distribution, the Anderson-Darling test [25] was applied. For this test, the hypothesis that the distribution is normal is rejected if the Anderson-Darling test statistic is greater than the critical value of 0.787 that corresponds to the conventional confidence threshold of 95 %. The Anderson-Darling test statistic calculated for the data set is 0.19, so the assumption that the data are normally distributed is accepted. The standard deviation of the distribution is 0.382. The mean is 0.029, and the standard deviation of the mean is 0.035, indicating that no significant bias is observed between the NIST results and the reference values.

## 7. Summary

Hearing aid directivity index measurements are done at NIST with the aid under test mounted on a standardized acoustical test manikin [13,14] to include the acoustical effects of a simulated median adult wearer on aid performance. By rotating the manikin in the horizontal plane and adjusting the vertical position of the loudspeaker used as the sound source, the directional response of the manikin-mounted aid is determined as a function of the source location defined in terms of azimuth and elevation angles. Directional response data used to determine the directivity factor and the directivity index are acquired with a standardized sampling method [15] that utilizes sound source locations distributed in a forty eight point semi-aligned zone array on an imaginary spherical surface surrounding the manikin. Expanded (k = 2) uncertainties of the directivity indices measured with the system range from 0.5 dB to 0.7 dB in the frequency range of one-third-octave bands with center frequencies from 500 Hz to 5000 Hz, and from 0.5 dB to 0.9 dB in the extended frequency range of one-third-octave bands with center frequencies from 200 Hz to 8000 Hz (see Table 4).

The capabilities of the measurement system were demonstrated over the frequency range of one-third-octave bands with center frequencies from 200 Hz to 8000 Hz through NIST participation in an interlaboratory comparison conducted between eight different laboratories of members of Working Group S3/WG48, Hearing Aids [23]. Directivity measurements were made for a total of six programmed memories in two different hearing aids and for the unaided manikin with the manikin right pinna circulated with the aids. Omnidirectional, cardioid, and bidirectional response patterns were measured. Results obtained with the NIST system were compared with the reference values of the comparison by calculating normalized deviations for all 119 values of the directivity factor measured (seven directional response patterns as a function of seventeen frequency bands). The absolute value of every normalized deviation is less than unity, which indicates that all the NIST measurement results agree with the reference values within the estimated uncertainty intervals. Furthermore, a histogram plot and the results of an Anderson-Darling test indicate that the normalized deviations are normally distributed, and that there is no significant bias between the NIST values and the reference values.

## Figures and Tables

**Table 1:** Forty eight sound source locations of the semi-aligned zone array for directivity measurements [15].

Elevation angle (degrees)	Azimuth angles (degrees)	Number of sound source locations
60	0, 60, 120, 180, 240, 300	6
30	0, 30, 60, 90, 120, 150, 180, 210, 240, 270, 300, 330	12
0	0, 30, 60, 90, 120, 150, 180, 210, 240, 270, 300, 330	12
−30	0, 30, 60, 90, 120, 150, 180, 210, 240, 270, 300, 330	12
−60	0, 60, 120, 180, 240, 300	6

**Table 2:** Elevation indices and power weights used to calculate the directivity factor/index from the data acquired at the five elevation angles of the forty eight point semi-aligned zone array [15].

Elevation angle (degrees)	Elevation index	Power weight (dimensionless)
60	1	0.1465
30	2	0.2241
0	3	0.2588
−30	4	0.2241
−60	5	0.1465

**Table 3:** Uncertainties of the directivity factors measured with the NIST system.

One-third octave band center frequency (Hz)	Standard uncertainties (%), all are Type B							Expanded (k = 2) uncertainty *U_N_* (%)
	*u_1_*	*u_2_*	*u_3_*	*u_4_*	*u_5_*	*u_6_*	*u_7_*	
200	1.3	1.3	2.7	2.7	2.7	2.7	2.7	12.6
250	1.3	1.3	2.7	2.7	2.7	2.7	2.7	12.6
315	1.3	1.3	2.7	2.7	2.7	2.7	2.7	12.6
400	1.3	1.3	1.3	2.7	2.7	2.7	2.7	11.7
500	1.3	1.3	1.3	2.7	2.7	2.7	2.7	11.7
630	1.3	1.3	1.3	2.7	2.7	2.7	4.2	13.4
800	1.3	1.3	1.3	2.7	2.7	2.7	4.2	13.4
1000	1.3	1.3	1.3	2.7	2.7	2.7	4.2	13.4
1250	1.3	1.3	1.3	2.7	2.7	2.7	5.5	15.1
1600	1.3	1.3	1.3	2.7	2.7	2.7	5.5	15.1
2000	1.3	1.3	1.3	2.7	2.7	2.7	5.5	15.1
2500	1.3	1.3	1.3	2.7	2.7	2.7	5.5	15.1
3150	1.3	1.3	1.3	2.7	2.7	2.7	5.5	15.1
4000	1.3	1.3	1.3	2.7	2.7	2.7	5.5	15.1
5000	1.3	1.3	1.3	2.7	2.7	2.7	7.0	17.4
6300	1.3	1.3	1.3	2.7	2.7	2.7	8.5	19.9
8000	1.3	1.3	1.3	2.7	2.7	2.7	10.1	22.7

**Table 4:** Uncertainties of the directivity indices measured with the NIST system.

One-third octave band center frequency (Hz)	Expanded (k = 2) uncertainty *U_N_* (dB)
200	0.5
250	0.5
315	0.5
400	0.5
500	0.5
630	0.5
800	0.5
1000	0.5
1250	0.6
1600	0.6
2000	0.6
2500	0.6
3150	0.6
4000	0.6
5000	0.7
6300	0.8
8000	0.9

## Footnotes

1 Certain commercial equipment, instruments, or materials are identified in this paper to foster understanding. Such identification does not imply recommendation or endorsement by the National Institute of Standards and Technology, nor does it imply that the materials or equipment identified are necessarily the best available for the purpose.
